# Cardiac implantable electronic device (CIED) infections are expensive and associated with prolonged hospitalisation: UK Retrospective Observational Study

**DOI:** 10.1371/journal.pone.0206611

**Published:** 2019-01-02

**Authors:** Fozia Zahir Ahmed, Catherine Fullwood, Mahvash Zaman, Ahmed Qamruddin, Colin Cunnington, Mamas A. Mamas, Jonathan Sandoe, Manish Motwani, Amir Zaidi

**Affiliations:** 1 Manchester Heart Centre, Manchester University NHS Foundation Trust, Manchester Academic Health Sciences Centre, Manchester, United Kingdom; 2 Manchester Academic Health Science Centre, Manchester University Hospitals NHS Foundation Trust, Manchester, United Kingdom; 3 Manchester Academic Health Science Centre, Research and Innovation, Manchester University Hospitals NHS Foundation Trust, Manchester, United Kingdom; 4 Centre for Biostatistics, Division of Population Health, Health Services Research and Primary Care, School of Health Sciences, Faculty of Biology, Medicine and Health, University of Manchester, Manchester, United Kingdom; 5 Department of Microbiology, Central Manchester University Hospitals NHS Foundation Trust, Manchester Academic Health Sciences Centre, Manchester, United Kingdom; 6 Keele Cardiovascular Research Group, Keele University, Stoke-on-Trent, United Kingdom; 7 Department of Medical Microbiology, Leeds Teaching Hospitals NHS Trust, Leeds, United Kingdom; 8 Leeds Institute of Biomedical & Clinical Sciences, University of Leeds, Leeds, United Kingdom; Kurume University School of Medicine, JAPAN

## Abstract

**Background:**

There are limited reports outlining the financial cost of treating cardiac implantable electronic device (CIED) infection outside the United States. This study aimed to determine the average treatment cost of CIED infection in a large UK tertiary referral centre and compared costs of different treatment pathways that are recognised in the management of CIED infection (early versus delayed re-implantation).

**Methods:**

We retrospectively analysed cost and length of stay (LOS) data for consecutive patients undergoing infected CIED extraction with cardiac resynchronization therapy (CRT-D [with defibrillator], CRT-P [with pacemaker]), implantable cardioverter-defibrillators (ICDs) and permanent pacemakers (PPMs).

**Results:**

Between January 2013 and March 2015, complete data was available for 84 patients (18 [21.4%] CRT-D, 24 [28.6%] ICDs and 42 [50.0%] PPMs). When all cases were considered the cost of infection ranged from £5,139 (PPM) to £24,318 (CRT-D). Considering different treatment strategies; 41 (48.8%) underwent CIED extraction and re-implantation during the same admission (early re-implant strategy (ER). 43 (51.2%) underwent extraction, but were then discharged home to be re-admitted for day-case re-implantation (delayed re-implant strategy (DR)). Median LOS was significantly shorter in DR compared to ER (5.0 vs. 18.0 days, p<0.001). The total cost of CIED infection episode was similar for both treatment strategies (median £14,241.48 vs. £14,741.70 including wearable defibrillator (Lifevest) and outpatient antibiotics costs, ER vs. DR; p = 0.491).

**Conclusion:**

CIED infections are expensive and associated with significant health-economic burden. When all device types were considered, a DR strategy is associated with reduced LOS without an increased cost penalty.

## Introduction

Rates of cardiac implantable electronic device (CIED) implantation have increased in the last decade. [1–5] However, rates of CIED infections are also increasing,[6, 7] and are associated with significant morbidity and mortality. [1, 8, 9] In 2011, the average cost of combined medical and surgical treatment for a single CIED infection in the USA ranged between $28,676 and $53,349.[8, 10] However, a detailed breakdown of the costs and health economic impact of treating CIED infection in the UK has not yet been reported. [10, 11] In addition to the cost of the device, there are multiple sources of potential expense; prolonged courses of antibiotics may be necessary, pacing-dependent patients may require temporary pacing, and patients with explanted implantable cardioverter defibrillators (ICDs) may need to remain in hospital for monitoring or be discharged with a wearable defibrillator (Lifevest), designed to detect and treat potentially life-threatening arrhythmias pending ICD re-implantation.[10]

A 2014 study reported the average cost of CIED infection in the UK to be £30,958 with a mean length of stay (LOS) of 29.9 days, although this study was small and measuring the cost of infection and was not the primary objective.[12] Different strategies are emerging for the management of these patients; some undergo a “single-stage” procedure where the infected device is removed and a new device is re-implanted during the same procedure. This approach potentially carries the potential risk of re-infection of the new device; accordingly, our centre has adopted a “two-stage” procedure for all patients, whereby a period of antibiotic therapy is completed following extraction, and a new device re-implanted at a later date. With such a strategy, some patients undergo re-implantation during the index hospitalisation (early re-implantation (ER), whilst others are discharged and subsequently return for day-case re-implantation at a later date (delayed re-implantation (DR). A DR strategy is often considered when patients have persistent signs of infection, including delayed healing of the index site of infection; or when clinically stable patients prefer to be managed in an outpatient setting whilst awaiting re-implantation. In the current study, we aimed to determine the average treatment cost of CIED infection in a large UK tertiary referral centre and to compare the cost of an ER vs. DR strategy.

## Methods

### Study design

Retrospective analysis of clinical case records of patients undergoing extraction for CIED infection at a single UK tertiary cardiothoracic centre. The principle outcomes were cost and length of stay. The study protocol, a data only study to examine data collected as part of routine clinical care, was reviewed and approved by the institutional review board (IRB) at Manchester University NHS Trust. In line with the Health Regulatory Authority (HRA) guidelines relating to data collected as part of routine care, written patient consent was not required as members of the care team were accessing data collected as part of usual clinical care.

### Setting

Manchester Heart Centre (MHC) serves a local population of 213,000 patients, and is a tertiary referral centre for CIED implantation and extraction for the wider conurbation of Greater Manchester and Lancashire. In 2016, 120 device extractions were performed at MHC.

### Participants

#### Inclusion and exclusion criteria

Patients aged >18 years who underwent CIED extraction for infection at MHC between 1^st^ January 2013 and 31^st^ March 2015. Patients who had the following device types were included: cardiac resynchronization therapy (CRT-D [with defibrillator], CRT-P [with pacemaker]), ICDs and permanent pacemakers (PPMs). Patients with implantable loop recorders and those with infection of temporary pacing systems were excluded.

#### Definitions

For the purposes of this analysis, a CIED infection episode was considered to start on the date of admission to MHC and end on the day of completion of antibiotic therapy for that episode (or discharge following re-implantation of a new device). CIED infections were sub-categorized, according to current UK guidelines, into the following groups; CIED-generator pocket infection (CIED-GPI), CIED-lead infection (CIED-LI), CIED-infective endocarditis (CIED-IE).[13]

Relapse of infection in the case of CIED-LI/IE was considered to have occurred if blood cultures became positive following initial resolution of clinical signs of infection, with blood cultures/device growing the same microorganism as the previous infective episode (confirmed with species identification), within 12 months of completing therapy. In the case of CIED-GPI, relapse was based on clinical evidence of infection occurring within 12 months of the initial infection episode.

#### Management strategy

All cases underwent “two-stage” management of their infection. Patients were classified into two groups: ER cases remained as inpatients for the duration of their treatment episode; DR cases had the infected device extracted and were discharged prior to re-implantation of a new device at a later date. In the DR group, the decision to receive outpatient antimicrobial therapy (OPAT), either parenteral or oral, was on advice of the microbiologists; guided by antimicrobial sensitivities and the trend in blood markers for infection.

### Variables and data sources

Baseline demographic data were collected on all patients. The Charlson comorbidity index, which estimates 10-year mortality according to comorbidity burden, was calculated.[14] The ongoing need for pacing, and/or defibrillator therapies, was evaluated for all cases. Data concerning type of device extracted, operative duration of extraction, length of inpatient stay (LOS) and type of device re-implanted were collected from an electronic pacing database. From these data, the (i) operative procedure cost (ii) device re-implantation cost and (iii) LOS were calculated and used to determine the total cost of CIED infection per patient. LOS comprised the total number of inpatient days related to the management of the episode of infection.

#### Cost analysis

The total cost of each CIED infection episode was calculated from the following formula:

Totalcost=procedurecost(extraction+re‑implantation)+costofinpatientstay+costoflifevest(ifapplicable)+costofOPAT(ifapplicable).

The procedure cost was calculated by adding the cost of materials used during the case (bar code analysis of discarded packaging) to the total catheter lab staff cost for the procedure (unit of laboratory time (ULT)). At MHC, one ULT costs £175, where one ULT equates to 30 minutes of staff time (covering the cost of a cardiologist, an anaesthetist, 2 nurses, a cardiac physiologist and a radiographer). Concerning the re-implantation procedure, the cost of the new device was based on the price paid locally to the manufacturer for that particular model, which is also recorded in the electronic patient records. The price of the discarded device was not included in calculations.

The cost of inpatient stay was calculated by multiplying the LOS (days) during the CIED infection episode by a daily bed cost of £350 per day per bed. Because of the significant additional cost, use of a Lifevest device was included separately. Cost was calculated from the duration that the Lifevest was issued to the patient, based on a monthly cost of £4,000 per patient.

Actual cost of inpatient antibiotic therapy was not prospectively recorded in the electronic database and therefore not available. However, details of OPAT were recorded as a daily tariff based on the cost of antibiotic therapy.

### Bias

We aimed to minimize bias by collecting data on consecutive patients, and to allow assessment of bias by describing why any eligible patients were excluded.

### Study size

The study size was opportunistic and based on the number of consecutive CIED extractions for infection undertaken at Manchester Heart Centre between 1^st^ January 2013 and 31^st^ March 2015.

### Quantitative variables and statistical analysis

Statistical analysis was performed using Prism version 6.0e and R version 3.2.4.[15] Demographic data are presented as median (IQR) or *N* (%). Group data were compared using a Mann-Whitney U-test or Fisher’s exact test, as appropriate. Financial data relating to the cost of treatment are presented as median (IQR), and compared using Mann Whitney U test. All tests were two-tailed and *P* <0.05 was considered statistically significant.

## Results

### Baseline demographics

Between 1^st^ Jan 2013 and 31^st^ March 2015, 106 patients underwent extraction for CIED-infection. Complete data relating to the cost of infection was available for 84 patients; 22 patients were excluded due to incomplete data. Baseline demographic data are presented in Table 1.

**Table 1:** Patient demographic data subdivided according to early (ER) or delayed (DR) re-implantation.

Demographics and clinical factors	Timing of CIED re-implantation		p
	Early re-implantation (n = 41)	Delayed re-implantation (n = 43)	
Median age, years (IQR)	73 (58.0–81.0)	69 (59.0–79.5)	0.594
Median Charlson index (IQR)	2 (0.0–3.0)	2 (1.0–3.5)	0.490
Median age-adjusted Charlson index (IQR)	4 (2.0–6.0)	4 (2.5–6.0)	0.712
Male sex, n (%)	33 (80.5)	36 (83.7)	0.780
Diabetes, n (%)	5 (12.2)	11 (25.6)	0.166
Heart failure (%)	20 (48.8)	25 (58.1)	0.512
CKD ≥3, n (%)	18 (43.9)	16 (37.2)	0.657
Congenital heart disease, n (%)	2 (4.9)	2 (4.7)	1.000
Prior CIED infection, n (%)	4 (9.8)	5 (11.6)	1.000
**Type of CIED infection**			
CIED-GPI	33 (80.5)	35 (81.4)	1.000
CIED-IE/LI	8 (19.5)	8 (18.6)	
**Presenting symptoms**			
Fever	11 (26.8)	10 (23.3)	0.804
Abscess	7 (17.1)	4 (9.3)	0.519
Erosion	12 (29.3)	16 (37.2)	0.488
Purulent discharge	16 (39.0)	17 (39.5)	1.000
Erythema	17 (41.5)	16 (37.2)	1.000
Raised blood markers for infection	17 (41.5)	25 (58.1)	0.190
Positive blood cultures	5 (12.2)	8 (18.6)	0.547
**CIED extracted**			
CRT-D	7 (17.1)	11 (25.6)	0.332
ICD	10 (24.4)	14 (32.6)	
PPM	24 (58.5)	18 (41.9)	
**Lifevest**			
CRT-D	0 (0.0)	9 (81.8)	
ICD	0 (0.0)	9 (64.3)	
PPM	0 (0.0)	0 (0.0)	
**Externalised pacemaker**			
CRT-D	0 (0.0)	1 (9.1)	
ICD	1 (10.0)	4 (28.6)	
PPM	5 (20.8)	4 (22.2)	

Forty-one (48.8%) patients underwent an ER strategy; a DR strategy was used in 43 (51.2%) patients. At the time of re-implant, 10 (11.9%) patients had an upgrade of their original device (PPM to CRT, n = 2; ICD to CRT-D, n = 8). Five (27.8%) patients whose original device was a CRT-D were re-implanted with an ICD only, one patient with a CRT-D was re-implanted with a CRT-P, and one patient was downgraded from an ICD to PPM at re-implant. A mechanical dilation tool (Evolution controlled rotational dilator system, Cook Medical) was used in 26 ER and 19 DR cases (p = 0.09).

Four patients had a relapse of CIED-GPI within 12 months of the index infection. A further two patients died during the index hospitalisation at day 31 and day 9 following PPM extraction (Case 1, aged 89 year old female with sepsis; Case 2, frail 90 year old male with multiple co-morbidities).

Table 2 shows the LOS and costs of CIED infection according to re-implantation strategy. These were analysed according to type of device extracted, as follows:

**Table 2:** LOS and cost of CIED infection according to re-implantation strategy.

	ER (n = 41)	DR (n = 43)	p
Median cost of index device extraction (cost of extracted device not included), £ (IQR)			
All	2,487.59 (1,184.10–3,633.49)	1,339.85 (900.54–3,229.94)	0.250
CRT-D	1,483.24 (1,094.43–3,447.86)	1,046.99 (916.70–4,200.12)	1.000
ICD	3,229.07 (1,880.18–5,161.52)	1,041.42 (922.79–2,113.38)	0.022
PPM	2,290.67 (1,000.74–3,549.99)	1,594.73 (882.36–3,706.37)	0.980
Median length of stay, days (IQR)			
All	18 (12–29)	5 (2–9)	<0.001
CRT-D	22 (1–27)	6 (5–13)	0.964
ICD	17 (12–23)	3 (2–5)	0.006
PPM	21 (15–30)	5 (1–8)	<0.001
Median cost of length of stay, £ (IQR)			
All	6,300.00 (4,200.00–10,150.00)	1,750.00 (525.00–2,975.00)	<0.001
CRT-D	7,700.00 (350.00–9,450.00)	2,100.00 (1,750.00–4,375.00)	0.964
ICD	5,775.00 (4,112.50–8,137.50)	875.00 (700.00–1,750.00)	0.006
PPM	7,350.00 (5,162.50–10,500.00)	1,750.00 (350.00–2,800.00)	<0.001
Median time from extraction to re-implant, days (IQR)			
All	14 (9–17)	33 (19–72)	<0.001
CRT-D	14 (8–20)	53 (36–91)	0.007
ICD	14 (8–16)	56 (20–72)	0.003
PPM	15 (12–16)	25 (15–33)	0.035
Re-implant device cost, £ (IQR)			
All	2,356.93 (1,412.69–12,150.00)	1,913.00 (1,181.85–9,374.74)	0.175
CRT-D	12,408.24 (10,941.00–12,996.97)	9,567.67 and 12,531.02*	0.659
ICD	12,996.88 (10,941.00–13,310.88)	9,374.74 (8,473.99–12,203.90)	0.015
PPM	1,914.61 (1,100.00–2,064.15)	1,181.85 (1,100.00–1,477.67)	0.173
Grand total cost of CIED infection (without Lifevest included), £ (IQR)			
All	14,241.48 (10,750.67–21,518.75)	12,252.68 (5,351.00–14,718.75)	0.003
CRT-D	20,049.00 (13,032.54–25,108.57)	16,026.74 (14,038.21–18,537.46)	0.659
ICD	22,077.18 (18,767.52–26,553.92)	12,472.91 (8,883.56–13,946.49)	<0.001
PPM	11,213.20 (8,634.09–15,881.56)	5,351.00 (3,155.02–10,873.82)	0.002
Lifevest cost, £ (IQR)			
All	N/A	10,000 (8,000–15,000)	
CRT-D	N/A	8,000 (8,000–24,000)	
ICD	N/A	12,000 (4,000–12,000)	
Grand total (including Lifevest cost), £ (IQR)			
All	14,241.48 (10,750.67–21,518.75)	14,739.74 (5,136.20–24,238.74)	0.491
CRT-D	20,049.00 (13,032.54–25,108.57)	24,315.80 (22,645.38–37,179.48)	0.056
ICD	22,077.18 (18,767.52–26,553.92)	18,174.94 (9,481.27–25,938.74)	0.192
PPM	11,213.20 (8,634.09–15,881.56)	5,136.20 (3,155.02–10,873.82)	0.001
Grand total (including Lifevest cost and outpatient antibiotics), £ (IQR)			
All	14,241.48 (10,750.67–21,518.75)	14,741.70 (5,139.14–24,253.93)	0.491
CRT-D	20,049.00 (13,032.54–25,108.57)	24,318.17 (22,727.97–37,193.48)	0.056
ICD	22,077.18 (18,767.52–26,553.92)	18,178.37 (9,481.27–25,938.74)	0.192
PPM	11,213.20 (8,634.09–15,881.56)	5,139.14 (3,158.26–10,891.56)	0.001

*Individual values due to small numbers

#### CRT-D

Of the 18 patients with an infected CRT-D, seven (38.9%) underwent an ER strategy and were re-implanted after a median of 14 days (IQR 8–20); eleven (61.1%) underwent a DR strategy and were re-implanted a median of 53 days later (IQR 36–91). Of these 11 DR patients, nine (81.8%) were discharged home with a Lifevest; none of these patients had any arrhythmia detected or received therapy. One patient who had an externalized pacemaker (and was thus not suitable for a Lifevest) requested to be managed as an outpatient pending device re-implant. Another, who had had an ICD for 13 years with no therapy, requested a downgrade to a CRT-P, and therefore did not receive a Lifevest.

#### ICD

Twenty-four patients had an infected ICD. Ten (41.7%) underwent an ER strategy a median of 14 days following extraction (IQR 8–16). Fourteen (58.3%) patients underwent a DR strategy and with re-implantation a median of 56 days later (IQR 20–72) (Table 2). Of these, nine (81.8%) patients received a Lifevest. Four patients who required an externalised pacemaker requested outpatient management so were unsuitable for a Lifevest, while one patient was downgraded to a pacemaker at re-implant.

#### Pacemakers

Twenty-four of 42 (57.1%) PPM patients underwent an ER strategy a median of 15 days later (IQR 12–16). An externalized temporary pacemaker was required in five (20.8%) of these cases following extraction. Eighteen (42.9%) PPM patients underwent a DRstrategy and were discharged with re-implantation a median 25 days later (IQR 15–33). An externalized temporary pacemaker was used in four (22.2%) cases.

#### Length of hospitalisation

When all device types were considered, total LOS was significantly shorter in patients managed via a DR strategy compared to an ER strategy (median 5 vs. 18 days, p<0.001). Those with either ICD or PPM in the DR group had significantly shorter LOS compared to similar patients in the ER group, [(ICD 3 vs. 17 days, p = 0.006) (PPM 5 vs. 21 days, p<0.001)]. Median LOS for patients with CRT-D was not significantly different (6 vs. 22 days, p = 0.964), however ER CRT-D comprised only seven patients with LOS located at both extremes of the range. Cost of hospitalization, attributable to bed costs, was significantly higher in ER patients (£6,300.00 vs £1,750.00; p<0.001) (Table 2). No patients required an intensive care unit (ICU) admission in either group.

### Cost analysis

For the total cohort of 84 cases, the median cost of a CIED extraction procedure was £1,729.95 (£942.41 – £3,588.90), and the cost of inpatient stay was £3,150 (£700.00 – £7,700.00).

#### Extraction procedure-related costs

Although the median cost of the CIED extraction procedure was higher for ER compared to DR, the difference was not statistically significant (£2,487.59 vs £1,339.85 respectively; p = 0.250) (Table 2). This cost takes into consideration the staff, equipment and facility-related costs for operative interventions.

#### Outpatient antibiotic therapy

Twenty-two (51.2%) of DR cases received OPAT prior to CIED re-implantation. Of these, one patient received intravenous OPAT; the remainder received oral treatment (flucloxacillin, n = 12; linezolid n = 5; other antibiotic n = 4). Median duration of outpatient treatment was 14 (12.5–14.0) days. Median cost was £3.47 (1.96–20.66). The staffing costs associated with administering IV antibiotics in the community were not calculated.

#### Cost of CIED infection episode according to device type

When all cases were considered, the cost of infection ranged £5,139 (PPM) to £24,318 (CRT-D).

The cost of the Lifevest increased the overall costs in the DR group by a median of £10,000.00; prior to the addition of Lifevest and OPAT costs, the cost of CIED infection episodes was significantly less in the ER group (£14,241.48 vs £12,252.68, p = 0.003). However, when the cost of the Lifevest and OPAT were taken into account, the total cost of CIED infection was similar in the ER and DR groups (£14,241.48 vs £14,741.70, respectively; p = 0.491). It should be noted that not all patients in whom a defibrillator was extracted (ICD or CRT-D) in the DR group received a Lifevest whilst awaiting re-implantation. Reasons for this included incompatibility with externalised pacemakers (1 CRT-D patient, 4 ICD patients), planned downgrade at re-implant (1 CRT-D patient, 1 ICD patient) and patient choice, i.e. patients unwilling to wear the Lifevest who accepted the short-term risk of untreated ventricular arrhythmias. When the groups were split according to type of CIED extracted (CRT-D, ICD or PPM), the total cost of CIED infection episode was significantly higher in ER cases compared to DR cases for PPM devices only (£11,213.20 vs £5,139.14; p = 0.001) (Table 2).

### Cost of episode according to type of CIED infection

Types of CIED infection are shown in Table 1. Because the number of cases of CIED-LI was relatively small (n = 3), the cases of CIED-IE and CIED-LI were combined for analysis. We examined cases according to the type of infection diagnosed; CIED-GPI (n = 68) and CIED-IE/LI (n = 16) (Table 3). The median total cost of CIED-GPI was £12,741.93, compared to £18,200.26 for cases of CIED-LI/IE (p = 0.105). The cost of operative intervention, relating to extraction of the infected device, was not significantly different between the two types of infection (CIED-GPI £2,121.49 vs £1,329.12; p = 0.214). LOS was significantly longer for cases of CIED-LI/IE compared to CIED-GPI (15 vs. 8 days; p = 0.029). Although the time from CIED extraction to re-implant was longer for cases of CIED-LI/IE compared to CIED-GPI, this was not statistically significant (30.0 vs. 16.0 days; p = 0.106).

**Table 3:** LOS and cost of CIED infection according to type of infection (CIED GPI vs. CIED LI/IE).

	CIED-GPI (N = 68)	CIED-LI/IE (N = 16)	p
Median cost of index device extraction (cost of extracted device is not included), £ (IQR)			
All cases	2,121.49 955.54–3,695.64	1,329.12 887.97–2,126.28	0.214
CRT-D	2,908.09 770.16–4,741.06	1,042.87 998.25–1,156.05	0.442
ICD	2,149.23 1,040.06–4,203.67	1,482.75 1,103.71–1,844.33	0.296
PPM	1,848.27 921.30–3,554.42	2,078.50 882.15–3,549.99	1.000
Median length of stay, days (IQR)			
All cases	8 (2–19)	15 (7–44)	0.029
CRT-D	9 (5–24)	5 (1–19)	0.631
ICD	4 (2–15)	8 (5–20)	0.293
PPM	10 (2–21)	41 (16–47)	0.005
Median cost of length of stay, £ (IQR)			
All cases	2,625.00 (700.00–6,562.50)	5,075.00 (2,537.50–15,400.00)	0.029
CRT-D	2,975.00 (1,750.00–8,225.00)	1,750.00 (350.00–6,475.00)	0.631
ICD	1,225.00 (700.00–5,075.00)	2,800.00 (1,750.00–7,000.00)	0.293
PPM	3,500.00 (700.00–7,350.00)	14,350.00 (5,512.50–16,275.00)	0.005
Median time from extraction to re-implant, days (IQR)			
All cases	16 (14–33)	30 (20–69)	0.106
CRT-D	33 (19–54)	100 (51–206)	0.449
ICD	17 (14–60)	20 (15–33)	0.867
PPM	15 (13–18)	30 (28–53)	0.019
Re-implant device cost, £ (IQR)			
All	2,356.93 (1,181.85–10,941.00)	2,553.76 (1,412.69–10,941.00)	0.628
CRT-D	10,941.00 (10,254.33–12,996.97)	12,408.24 and 12,531.02	0.883
ICD	12,150.00 (9,374.74–13,263.00)	10,941.00 (10,549.43–11,270.20)	0.559
PPM	1,181.85 (1,100.00–1,996.20)	1,412.69 (1,288.15–1,996.20)	0.462
Grand total cost of CIED infection before life vest, £ (IQR)			
All	11,819.42 (7,878.16–15,585.93)	15,996.37 (13,722.47–19,505.43)	0.028
CRT-D	17,869.81 (13,844.91–21,911.97)	15,134.11 (14,161.05–16394.30)	0.574
ICD	13,781.73 (11,262.33–21,200.92)	15,431.83 (14,455.88–19,269.87)	0.388
PPM	9,324.07 (5,151.98–11,686.68)	18,270.59 (8,860.31–20,529.31)	0.022
Lifevest cost			
All cases	8,000 8,000–12,000	4,000 16,000 40,000*	
CRT-D	8,000 8,000–16,000	16,000 & 40,000	
ICD	12,000 7,000–12,000	4,000	
Received externalized pacemaker	13	2	
Grand total cost of CIED infection, £ (IQR)			
All	12,741.93 (7,196.04–22,707.29)	18,197.32 (13,963.76–22,914.12)	0.105
CRT-D	23,647.89 (20,575.91–28,824.90)	24,761.87 (16,683.11–37,500.00)	0.959
ICD	21,306.86 (14,414.86–26,218.39)	17,431.83 (15,257.15–21,468.61)	0.852
PPM	9,324.07 (5,088.22–11,687.68)	18,270.59 (8,860.31–20,529.31)	0.018
Grand total cost of CIED infection with antibiotics, £ (IQR)			
All	12,741.93 (7,196.04–22,707.29)	18,200.26 (13,963.76–22,914.12)	0.105
CRT-D	23,675.89 (20,610.21–28,824.90)	24,767.19 (16,683.11–37,507.98)	<0.001
ICD	21,306.86 (14,416.33–26,218.58)	17,434.76 (15,257.15–21,473.02)	<0.001
PPM	9,324.07 (5,089.69–11,686.68)	18,270.59 (8,908.31–20,529.31)	<0.001

*Only three patients in this group receive a Lifevest, therefore statistical test not appropriate due to low number of cases.

## Discussion

This is the largest study to evaluate the cost of CIED infection in the UK. We found that a DR strategy, whereby the patient is discharged after CIED extraction with re-implantation at a later date (on a daycase basis), is feasible and associated with no overall cost penalty, taking into account all device types. Lower costs for DR were seen for PPM infections, due to reduced LOS. There was a trend towards higher overall cost in the CRT-D group managed using a DR strategy and also higher overall cost for CIED-LI/IE compared to CIED-GPI cases, though these were not statistically significant.

US data indicates that the combined medical and surgical cost for treatment of CIED infection has increased by 47% over the last 20 years.[1] While the bulk of expense may be attributed to the cost of the re-implanted device and procedure, there are other significant expenses that contribute, including diagnostic investigations, medical (e.g. intravenous antibiotics) and surgical intervention, and prolonged hospital stay. Sohail *et al*. reported the mean adjusted cost of admissions related to CIED infection to range from $28,676 to $53,349 depending on the type of device infected (PPM- $28,676, CRT-P- $39,410, ICD- $47,543, CRT-D- $53,349), where almost half of the incremental cost was attributable to stay on an intensive care unit.(8) In another study, compared to CIED patients without infection, the incremental healthcare expenditure for patients with CIED infection occurring within 12 months of index implantation requiring in- or out-patient extraction for infection was $45,291, and $279,744 for those with severe sepsis requiring in-patient extraction.[16]

Thus far, there have been only limited UK data.[17] One prior study of 30 cases in 2014 reported the UK cost of CIED infection to be approximately £30,958.40, similar to US studies, [8, 11, 12] but higher than we report in the current study (median £14,491.59). This may be partly explained by the fact that, our population contained a larger number of lower power devices (PPM; n = 42) compared to the previous analysis (PPM; n = 8). Moreover, in the study by Ahsan *et al*, calculation of post-extraction care included critical care (level 3) stay, compared to coronary care unit (level 2) in the current study.Finally, Ahsan *et al*. included the cost of inpatient antimicrobial treatment, which although this does not constitute the bulk of additional cost, is a limitation of our study.[12] European data is also sparse. In a single centre study, which included 7 pacemaker infections, Kuehn *et al*. reported that the mean additional hospital costs for infected cases was €7091.[17] More recently, a retrospective analysis of German health claims data has indicated that the incremental healthcare expenditure for patients with CIED infection ranged from €31,493 for denovo infections to €59,419 for major infections.[18]

When all cases were considered, median (IQR) LOS was 9 (2–22) days for all CIED infections. The PPM group had the highest median LOS at 15 (5–24) days. Interestingly, we report longer LOS with low energy devices (PPM) compared to ICD/CRT-D, although this was not statistically significant (15 days vs. 8 days; p = 0.571). US data indicates that the mean adjusted LOS for CIED infection ranged from 15.5 to 24.3 days depending on the type of device (PPM 15.5, CRT-P 24.3, ICD 18.8, CRT-D 17.1 days). The incremental LOS with infection ranged from 9.4 to 18.2 days.[8]

Very early re-implant strategies, with CIED re-insertion 72 hours post-extraction, have also been described,[19] although this is not currently practiced at our institution. In the current study, median time from extraction to re-implant for ER cases was 14 days, compared to 33 days in the DR group (p<0.001).

We have demonstrated that a DR strategy is feasible, and that whilst the costs per individual range considerably, the overall cost for adoption of such a strategy is cost neutral, even taking into account the cost of a Lifevest for ICD patients. The authors believe that the additional benefit of LOS reduction (e.g. the ability to use hospital beds to treat other patients) makes a strong case for using the DR strategy in the UK.

### Limitations

Although the principle indication for being stratified to a DR treatment strategy is usually persistent or slow healing infection, there is the possibility of some such patients being in the ER group due to other circumstances. We acknowledge this as a limitation of a retrospective observational study but, as this was a single-centre study with fairly homogenous clinical practice amongst our specialists, we expect the impact of this limitation to minimal. Furthermore, as cases of device infection are discussed in a dedicated MDT meeting, significant variation in usual care is limited.

Our study has several limitations that may underestimate the cost of infection. As some patients had re-implantation performed at their local hospitals, the number of cases included was reduced from 106 to 84 and thus some of the subgroups contained small numbers. Costs incurred via diagnostic procedures and some costs relating to treatment of infection (e.g. delivering intravenous antibiotics) were not included, although we do not feel that these represented a major confounding factor. Regarding LOS, we report the longest LOS in the PPM cohort, however this is likely to be underestimated as 20 PPM patients were transferred as inpatients from their local hospitals for extraction, and we do not include the additional LOS incurred at those hospitals.

The costs quoted are based on prices paid by our centre, however these may vary across the UK. The ULT used to calculate staff and logistical costs for each procedure was derived from a local model, and we recognise that this cost may not be generalizable as the make-up of the catheter lab team may vary between centres; indeed, in some hospitals CIED extraction is performed by cardiac surgeons in an operating theatre. Finally, the cost analysis in this study only examines the cost of inpatient treatment and does not take into consideration any outpatient appointments, investigations or previous hospitalisations relating to a particular episode of infection.

## Conclusion

CIED infections are expensive and associated with significant health economic burden due to prolonged hospitalization, procedural and device costs. Consideration of different approaches to management of CIED infection, such as a DR strategy for those patients with delayed healing or infection that is slow to fully resolve, may help to mitigate the cost of CIED infection and warrants further prospective evaluation provided the approaches are as efficacious and not associated with a higher rate of adverse events.
