# Correction: Cardiac implantable electronic device (CIED) infections are expensive and associated with prolonged hospitalisation: UK Retrospective Observational Study

**DOI:** 10.1371/journal.pone.0213682

**Published:** 2019-03-06

**Authors:** Fozia Zahir Ahmed, Catherine Fullwood, Mahvash Zaman, Ahmed Qamruddin, Colin Cunnington, Mamas A. Mamas, Jonathan Sandoe, Manish Motwani, Amir Zaidi

## Notice of republication

This article was republished on February 22, 2019 to remove a Supporting Information file that was incorrectly included in the originally published article. Please download this article again to view the correct version.
